# Impact of Critical Illness Insurance on the Burden of High-Cost Rural Residents in Central China: An Interrupted Time Series Study

**DOI:** 10.3390/ijerph16193528

**Published:** 2019-09-20

**Authors:** Lu Li, Junnan Jiang, Li Xiang, Xuefeng Wang, Li Zeng, Zhengdong Zhong

**Affiliations:** School of Medicine and Health Management, Huazhong University of Science and Technology, Wuhan 430030, China; lltj@hust.edu.cn (L.L.); jiangjunnan@hust.edu.cn (J.J.); M201775310@hust.edu.cn (X.W.); M201875307@hust.edu.cn (L.Z.); M201875308@hust.edu.cn (Z.Z.)

**Keywords:** interrupted time series study, critical illness insurance, high-cost, out-of-pocket, reimbursement ratio, financial burden

## Abstract

Critical illness insurance (CII) in China was introduced to protect high-cost groups from health expenditure shocks for the purpose of mutual aid. This study aimed to evaluate the impact of CII on the burden of high-cost groups in central rural China. Data were extracted from the basic medical insurance (BMI) hospitalization database of Xiantao City from January 2010 to December 2016. A total of 77,757 hospitalization records were included in our analysis. The out-of-pocket (OOP) expenses and reimbursement ratio (RR) were the two main outcome variables. Interrupted time series analysis with a segmented regression approach was adopted. Level and slope changes were reported to reflect short- and long-term effects, respectively. Results indicated that the number of high-cost inpatient visits, the average monthly hospitalization expenses, and OOP expenses per high-cost inpatient visit were increased after CII introduction. By contrast, the RR from BMI and non-reimbursable expenses ratio were decreased. The OOP expenses and RR covered by CII were higher than those uncovered. We estimated a significant level decrease in OOP expenses (*p* < 0.01) and rise in RR (*p* < 0.01), whereas the slope decreases of OOP expenses (*p* = 0.19) and rise of RR (*p* = 0.11) after the CII were non-significant. We concluded that the short-term effect of the CII policy is significant and contributes to decreasing OOP expenses and raising RR for high-cost groups, whereas the long-term effect is non-significant. These findings can be explained by increasing hospitalization expenses, many non-reimbursable expenses, low coverage for high-cost groups, and the unsustainability of the financing methods.

## 1. Introduction

Healthcare costs are concentrated among a small group of the most expensive patients. This group is identified as “high-need, high-cost” patients [1,2]. Previous studies have shown that the top 10%, top 5%, and top 1% high-cost patients roughly account for, respectively, 68%, 55%, and 24% of costs within a given year [3]. A few studies from the United States, European countries, Hong Kong, and Taiwan, all with different health insurance schemes and healthcare delivery systems, have presented similar findings [4,5,6,7,8]. These findings suggest that high-cost patients are a logical group to seek for cost-reduction financial support; caring for this group is an urgent priority [9].

Many insurance plans pursue this logic, and programs for “high-need, high-cost” patients have been developed [10,11]. In China, critical illness insurance (CII) is an insurance plan aimed at providing further reimbursement of high medical expenses associated with critical illness, based on the basic medical insurance (BMI) system. The initial objective of CII was to protect those “high-cost” patients from health expenditure shocks for the purpose of mutual aid and to solve the problems of illness-caused poverty. By May 2014, more than half of the rural regions in China had introduced the CII program [12]. An insured rural resident who incurs high medical costs can first receive reimbursement through BMI. Subsequently, the patients whose out-of-pocket (OOP) expenses still exceed the premium (usually the local income per capita) can obtain additional reimbursement [13]. To answer whether the CII policy is reaching its original objective, following the high-cost group is crucial.

The existing literature has mainly focused on describing and analyzing the policy design of CII [14,15,16]. Certain surveys have evaluated the effect of CII from the perspectives of financial burden [17,18] and equity [19]. These studies have targeted all beneficiaries with cross-sectional data. Research assessing the effect of CII on the high-cost group has not yet been conducted, nor has research using longitudinal data. Using the longitudinal BMI claim dataset of seven years, the present study adopted interrupted time series (ITS) analysis to evaluate the impact of CII on the burden of the high-cost group in central rural China. 

## 2. Materials and Methods 

### 2.1. Study Setting and the CII Policy

**Figure 1:**
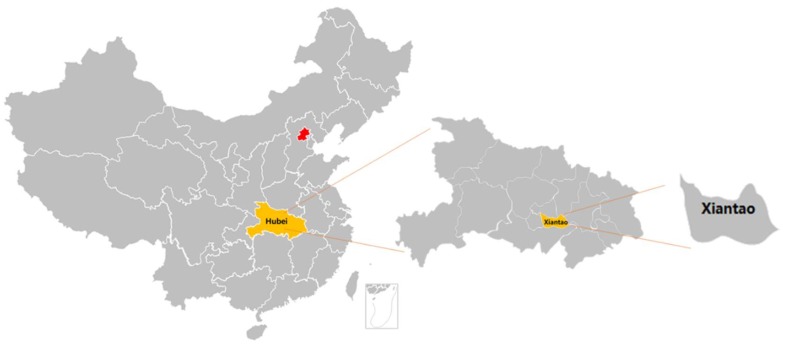
Location of sample city.

Xiantao City was selected as the research sample for two reasons. Geographically, the city is located in the central part of Hubei Province in China (Figure 1). In economic terms, Xiantao is representative of China in terms of per capita GDP. In 2013, the national per capita GDP was US$6995, and the per capita GDP in Xiantao was US$6854. Xiantao has a total area of 2538 km^2^ and had a total population of 1,563,500 at the end of 2016. The annual net income of rural residents in Xiantao was US$1005 in 2010 and US$2329 in 2016.

In May 2013, the Xiantao government enacted the CII policy for the first time. Table 1 shows the details of the reimbursement system and its changes before and after the CII policy was implemented. Before the introduction of the CII policy, patients received reimbursement from BMI. The reimbursement methods differ between healthcare services in- and out-of-county. Patients seeking services in-county can apply for lower thresholds and a higher policy reimbursement ratio (PRR) for the same medical condition. In 2011, the local government abolished the deductible for seeking health services at primary hospitals in-county and raised the cap line from US$8070 to US$16,140. After the introduction of the CII policy, the local government raised the threshold of BMI. The reimbursement system includes BMI and CII, both of which share the same fund pool and reimbursement list. Expenses out of the scope of the list are non-reimbursable. The deductible of CII was US$1291, set with reference to per capita annual income. PRR was 50% for OOP payments within the range of US$1291–US$4842, 60% for payments of US$4842–US$8070, and 70% for OOP payments over US$8070. 

For instance, if a patient’s medical expenses totaled US$19,368, assuming that all were included in the benefit package and could be reimbursed, the BMI could cover US$16,140. Before the CII policy was implemented, the reimbursement and the OOP expenses would have been US$16,140, and US$3228, respectively. After the CII policy was implemented, the OOP expenses became US$2260 (19,368 − 16,140 − (3228 − 1291) × 50%).

### 2.2. Data Collection

Data were extracted from the BMI hospitalization database of Xiantao City from January 2010 to December 2016, covering an observation period of seven years. High-cost visits, which were the top 10% of total hospitalization expenditures of records each year, were used in this study by referring to the criteria of previous studies [20,21]. A total of 77,757 hospitalization records were included in our analysis. During the study period 2010–2016, the total population and the aged proportion of the population grew, the male to female ratio increased, and the urbanization rate increased by 7.2% within the seven years. In general, the population structure was relatively stable.

### 2.3. Study Variables

To explore the changes in the quantity, structure, and expenses of high-cost inpatient visits before and after the CII policy, we accessed several key variables. The total numbers of high-cost inpatient visits were used to compare changes in quantity. The proportion of high-cost inpatients visits covered by CII was used to assess the scope of beneficiaries. Considering the differentiated reimbursement policy for medical treatment in- or out-of-county mentioned above, the proportion of high-cost inpatient visits in-county was assessed to compare the difference with out-of-county visits before and after the CII policy. The hospitalization expenses and OOP expenses of high-cost inpatient visits were also assessed. CII and BMI reimbursement ratio (RR) and non-reimbursable expenses ratio per high-cost inpatient visit were further measured.

The OOP expenses and RR were the two main outcome variables. For insured patients, part of their total medical expenses can be reimbursed by the insurance system; these expenses are called reimbursements. The remaining costs paid by such patients are called OOP expenses. In this study, the total medical expenses included all the categories of direct medical costs, typically including costs of treatment, laboratory tests, accommodation, materials, and drugs. Direct non-medical costs, such as transportation, and indirect costs, such as work loss, were not included. RR was defined as reimbursements divided by total medical expenses. With the function of reflecting the economic burden of patients and support from the reimbursement system, the OOP expenses and RR have been widely used in similar research [22,23,24,25].

Cost data inflation were adjusted with annual net income. Equivalent US$ has been reported using the average annual exchange rate. 

### 2.4. Statistical Analysis

ITS analysis with a segmented regression approach [26] was adopted. ITS analysis is regarded as one of the strongest quasi-experimental methods that can be used to capture the longitudinal effects of policy interventions when a control group is lacking [27,28]. The segmented regression model was employed to detect the level and trend of outcome variables before the implementation of the CII policy, and the changes in level and trend after the implementation of the policy. Level change indicates the change at the moment of intervention. Slope change indicates a long-term effect of the policy. The regression model is as follows:

(1) $$Y_{t}=\beta_{0}+\beta_{1}*time_{t}+\beta_{2}*intervention_{t}+\beta_{3}*time\;after\;intervention_{t}+\varepsilon_{t},$$

where $Y_{t}$ represents the mean value of each evaluation indicator, and ${\mathrm{time}}_{t}$ represents a continuous variable indicating time in years at time *t* from the start of the observation period.${\;\mathrm{Intervention}}_{t}$ represents an indicator for time *t* occurring before (intervention = 0) or after (intervention = 1) the implementation of the CII policy. May 2013 was defined as the start time of the intervention. ${\mathrm{Time}\;\mathrm{after}\;\mathrm{intervention}}_{t}$ represents a continuous variable counting the number of months after the intervention at ${\mathrm{time}}_{t}$, coded 0 before the CII (from January 2010 to April 2013) and coded 1–44 after the CII (from May 2013 to December 2016). Detailed information is presented in Appendix A.

$\beta_{0}$ estimates the baseline level of the outcome at time 0. $\beta_{1}$ estimates the change that occurs per month before the CII (baseline slope). $\beta_{2}$ estimates the immediate level change after the CII (level change after the CII). $\beta_{3}$ estimates the change in the trend after the CII (slope change after the CII), compared with the monthly trend before the CII. $\varepsilon_{t}$ is an error term. 

The Durbin–Watson test was conducted to detect autocorrelations. The Cochrane–Orcutt auto-regression procedure was used to correct for first-order serially correlated errors when needed. All analyses were performed using STATA 12.0 (STATA Corp., College Station, TX, USA). 

## 3. Results

### 3.1. Changes of Indicators before and after the Implementation of the CII Policy

Table 2 shows that after the introduction of the CII policy, the total high-cost inpatient visits increased, and the proportion of high-cost inpatient visits covered by the CII accounted for 44% of monthly visits on average. The proportion of in-county inpatient visits increased and accounted for 86.9% of monthly visits on average. The average monthly hospitalization expenses and OOP expenses per high-cost inpatient visit all increased. By contrast, the average monthly BMI RR and non-reimbursable expenses ratio per high-cost inpatient visit decreased.

### 3.2. Trends of Out-of-Pocket Expenses and Reimbursement Ratio (RR) in 2010–2016

**Figure 2:**
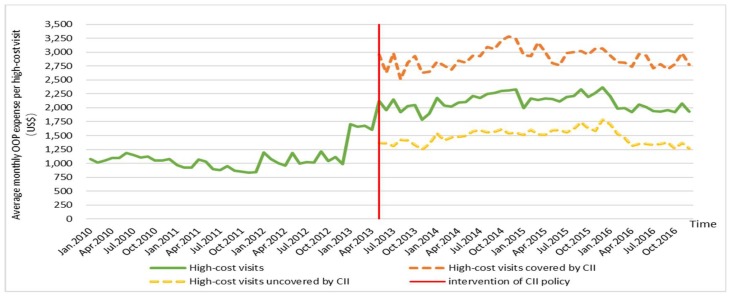
Trends of out-of-pocket (OOP) expenses in 2010–2016.

**Figure 3:**
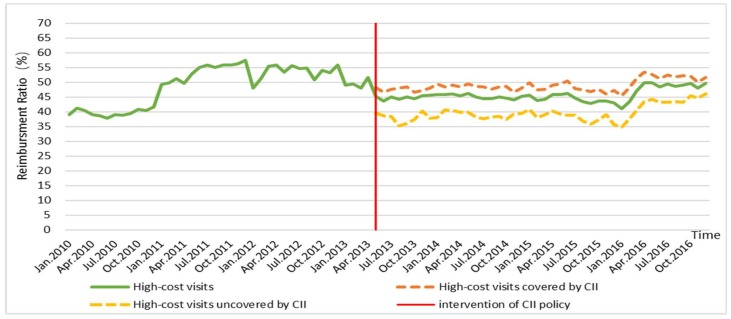
Trends of reimbursement ratio (RR) in 2010–2016.

Figure 2 and Figure 3 present the time series of OOP expenses and RR of high-cost visits from 2010 to 2016. Figure 2 displays a rough upward tendency of average monthly OOP expenses per high-cost visit, which increased from US$1081 to US$1927. Figure 3 shows the process of average monthly RR per high-cost visit, where it first descended and then slightly rose. The average monthly OOP expenses per visit out-of-county were higher than those in-county, whereas the situation of RR was the reverse. After the implementation of the CII policy, the average monthly OOP expenses and RR per visit of high-cost visits covered by the CII were higher than those uncovered by the CII. That is, those covered by CII indeed had a larger financial burden than those uncovered and those who received additional help from the CII policy.

### 3.3. Impact of the Critical Illness Insurance Policy

Table 3 presents the impact of the CII policy on OOP expenses and RR. Before the introduction of CII, an increasing trend existed in the OOP expenses. After the CII was implemented, a significant abrupt level change was observed, with an increase of US$682.6 (*p* < 0.01, 95% confidence interval (CI) = 446 to 919.2). The slope change showed a decrease of US$9.1, but was not statistically significant (*p* = 0.19, 95% CI = −22.6 to 4.4). A decrease trend occurred in the RR during the pre-implementation period. During the post-implementation period, a significant abrupt level change was noted, with a decrease of 7.9% (*p* < 0.01, 95% CI = −12.1 to −3.7). The slope change showed an increase of 0.3%, but was not statistically significant (*p* = 0.11, 95% CI = −0.7 to 0.1). These changes can be seen in Appendix B.

## 4. Discussion

This study described an overview of the changes in the OOP expenses and RR of high-cost inpatient visits among rural residents after the implementation of the CII policy in May 2013. Through ITS analysis, we estimated a statistically significant decrease in OOP expenses (*p* < 0.01) and rise in RR (*p* < 0.01) for the high-cost group after the introduction of CII. This finding indicates that CII has an immediate impact on easing the financial burden of this group. In the short term, the CII is an effective policy because it provides further protection beyond BMI. This result is in line with those shown in previous studies [17,18,19].

However, the decrease trend of OOP expenses (*p* = 0.19) and the rising trend of RR (*p* = 0.11) after CII were not statistically significant. Therefore, the CII policy cannot effectively ease the burden of high-cost rural residents in the long term. To the best of our knowledge, our study is the first quantitative research to investigate the long-term effects of the CII policy on financial burden.

Several possible reasons may account for these invalid long-term effects. 

First, the hospitalization expenses greatly increased. The uncovered medical expenses were a considerable element of total hospitalization expenses. According to the research of Pavón-León et al., uncovered medical expenses rise from 12.8% to 27.5% as total medical costs increase [29]. The high-cost group usually needs additional medical consumable materials and drugs that are not included in the reimbursement list. This part of the medical expenses is ignored and not reimbursed by either BMI or CII. We found that non-reimbursable expenses accounted for more than a quarter of the total expense for high-cost inpatient visits, even after the implementation of the CII policy. 

Although the CII policy helps keep inpatient visits in-county, we found nearly 15% of high-cost inpatient visits were out-of-county. However, reimbursement rules were typically not that generous for out-of-county visits, thus aggregating the financial burden of the high-cost group. 

Moreover, the high-cost beneficiaries of the CII policy are limited. In this study, no more than half high-cost visits were found to be covered by the CII policy; and the OOP expenses covered by the CII were higher than those uncovered. On the one hand, the population insured with CII consisted of patients whose OOP expenses still exceeded the deductible after being reimbursed by BMI. The medical costs of this group are much higher than those of the uninsured. On the other hand, only costs within the scope of the CII can be reimbursed, and the CII has the same limited reimbursement list as BMI. In addition, the PRR is not high enough, and thus the proportion of reimbursement was insufficient to make up for the gap of medical costs between the insured and uninsured groups. These factors may explain why the OOP expenses of the population insured with CII can be higher than those uninsured. 

The CII fund comes from the BMI fund pool, giving rise to great pressure on the BMI fund pool over time and thus comprising the sustainability of the safeguard. Xiantao City has lowered the BMI PRR to alleviate fund pressure, which explains why the BMI effective RR decreased by nearly 5% after the introduction of the CII policy.

To further ease the financial burden of high-cost beneficiaries of the CII policy in the long run, many rules can be improved. We suggest considering other aspects, such as cost of critical illness at the local level, when setting deductibles, not just going by per capita income of rural residents. An additional and specific reimbursement list which contains commonly used drugs and materials for the high-cost group is needed. Raising extra funds for the CII policy through diversified financing channels, such as government finance, charity, and civil administration, would be a feasible way to ensure sustainability. 

The limitations of this study should be mentioned. We only conducted the study in one city in central China. However, as the CII policy operates at the city level, we acquired complete and high-quality data focusing on a single city. The synergistic effects of other health policies, such as medical financial assistance, were not considered. 

## 5. Conclusions

This study adopted ITS analysis to evaluate the impact of the CII on the burden of high-cost group in central rural China with a seven-year longitudinal dataset. The short and long-term effects were investigated. The results indicated that the short-term effect was significant and contributed to decreasing the OOP expenses and raising the RR of the high-cost group. By contrast, the long-term effect was non-significant. Explanations such as increasing hospitalization expenses, large non-reimbursable expenses, low coverage for the high-cost group, and unsustainability of financing methods were also explored. Suggestions have been made to improve the CII policy and alleviate the financial burden of the high-cost group, for example, setting the deductible with reference to the cost of critical illness at the local level, developing an additional and specific reimbursement list, and raising extra funds for the CII policy through diversified financing channels. Further research is necessary to evaluate the long-term effects of the CII policy with a wide scope.

## Figures and Tables

**Table 1:** Policy changes before and after the critical illness insurance (CII) policy was implemented.

**Before the CII Policy Was Implemented**							
**BMI (Year 2010)**				**BMI (Year from 2011)**			
**Deductible**		**PRR ^1^**	**Cap**	**Deductible**		**PRR ^1^**	**Cap**
(US$)		(%)	(US$)	(US$)		(%)	(US$)
A1 ^2^	16	80	8070	A1 ^2^	0	90	16,140
A2 ^3^	32	60		A2 ^3^	32	80	
B ^4^	81	40		B ^5^	81	60	
**After the CII Policy was Implemented**							
**BMI**				**CII**			
**Deductible**		**PRR ^1^**	**Cap**	**Deductible ^5^**	**Expenses**	**PRR ^1^**	**Cap**
(US$)		(%)	(US$)	(US$)	(US$)	(%)	(US$)
A1 ^2^	16	90	16,140	1291	1291–4842	50	No cap
A2 ^3^	81	60			4842–8070	60	
B ^4^	129	50			>8070	70	

^1^ PRR: Policy Reimbursement ratio. ^2^ A1: Seeking health services at primary hospital in-county. ^3^ A2: Seeking health services at secondary hospital in-county. ^4^ B: Seeking health services out-of-county. ^5^ Deductible is defined here as those medical expenses within the scope of insurance that exceed the threshold after being reimbursed by basic medical insurance (BMI).

**Table 2:** Key indicators of policy effects in Xiantao.

Variables	Implementation of the CII Policy	
	Before (Mean ± SD ^1^)	After (Mean ± SD)
Total number of high-cost inpatient visits	28,448	49,309
Proportion of high-cost inpatients visits covered by CII (%)	0	44.0 ± 3.2
Proportion of high-cost inpatients visits in-county (%)	60.9 ± 34.1	86.9 ± 21.6
Hospitalization expenses of high-cost inpatient visits (US$)	2155.6 ± 438.6	3863.5 ± 207.5
OOP expenses of high-cost inpatient visits (US$)	1089.6 ± 216.8	2100.4 ± 140.1
CII Reimbursement ratio per high-cost inpatient visit (%)	0	8.0 ± 2.8
BMI Reimbursement ratio per high-cost inpatient visit (%)	42.6 ± 16.2	38.8 ± 2.1
Non-reimbursable expenses ratio per high-cost inpatient visit (%)	34.1 ± 28.1	25.4 ± 1.5

^1^ SD: Standard Deviation.

**Table 3:** Interrupted time series (ITS) analysis results of policy effect on OOP expenses and reimbursement ratio (RR) in Xiantao.

Variables	Value (SE ^1^)	t-Value	*p*-Value	95% CI	DW ^2^
**OOP (US$)**					2.2
β0: Baseline level	918.1 (111.1)	8.3	<0.01 ^3^	(697.0, 1139.2)	
β1: Baseline slope	10.7 (4.6)	2.3	0.02	(1.6, 19.9)	
β2: Level change after CII policy	682.6 (118.9)	5.7	<0.01 ^3^	(446.0, 919.2)	
β3: Slope change after CII policy	−9.1 (6.8)	−1.3	0.19	(−22.6, 4.4)	
**Reimbursement Ratio (%)**					2.2
β0: Baseline level	41.5 (3.0)	13.8	<0.01 ^3^	(35.5, 47.5)	
β1: Baseline slope	0.3 (0.1)	2.7	0.01	(0.1, 0.6)	
β2: Level change after CII policy	−7.9 (2.1)	−3.7	<0.01 ^3^	(−12.1, −3.7)	
β3: Slope change after CII policy	−0.3 (0.2)	−1.6	0.11	(−0.7, 0.1)	

^1^ SE: Standard Error. ^2^ DW: Durbin–Watson test value. ^3^ *p* < 0.05.

**Figure A1:**
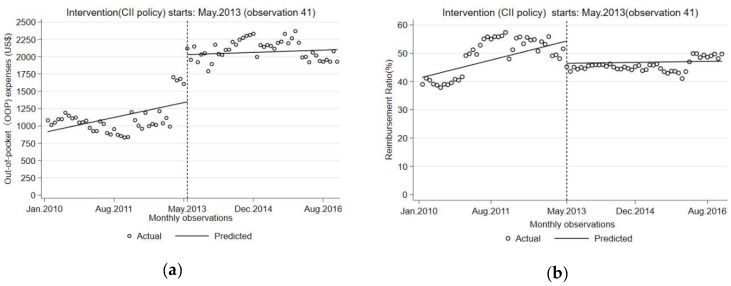
Graphical demonstration of interrupted time series (ITS) analysis. (**a**) CII policy effect on OOP expenses; (**b**) CII policy effect on Reimbursement Ratio (RR).

## Appendix A

**Table A1:** Detailed information of interrupted time series (ITS) analysis, medical expenses and medical services utilization.

Year	Observation	Time	Intervention	Time after Intervention	THE ^1^ (US$)	OOP ^2^ (US$)	RR ^3^ (%)	Inpatient Visits
Jan. 2010	1	1	0	0	1770.3	1080.5	39.0	359
Feb. 2010	2	2	0	0	1724.1	1014.6	41.2	261
Mar. 2010	3	3	0	0	1757.8	1047.1	40.4	475
Apr. 2010	4	4	0	0	1798.5	1095.9	39.1	480
May 2010	5	5	0	0	1791.5	1098.6	38.7	444
June 2010	6	6	0	0	1913.7	1190.0	37.8	455
July 2010	7	7	0	0	1881.4	1147.2	39.0	429
Aug. 2010	8	8	0	0	1812.8	1108.4	38.9	436
Sep. 2010	9	9	0	0	1852.1	1120.5	39.5	433
Oct. 2010	10	10	0	0	1771.0	1048.5	40.8	345
Nov. 2010	11	11	0	0	1766.0	1051.7	40.4	537
Dec. 2010	12	12	0	0	1840.6	1074.2	41.6	769
Jan. 2011	13	13	0	0	1909.8	971.2	49.1	398
Feb. 2011	14	14	0	0	1841.5	924.3	49.8	329
Mar. 2011	15	15	0	0	1895.4	924.7	51.2	599
Apr. 2011	16	16	0	0	2111.4	1065.3	49.5	562
May 2011	17	17	0	0	2182.2	1029.5	52.8	627
June 2011	18	18	0	0	1994.8	896.5	55.1	750
July 2011	19	19	0	0	1982.1	875.9	55.8	789
Aug. 2011	20	20	0	0	2116.7	953.2	55.0	785
Sep. 2011	21	21	0	0	1975.2	872.2	55.8	719
Oct. 2011	22	22	0	0	1930.3	853.3	55.8	655
Nov. 2011	23	23	0	0	1899.0	833.1	56.1	759
Dec. 2011	24	24	0	0	1964.4	838.3	57.3	926
Jan. 2012	25	25	0	0	2304.4	1199.2	48.0	608
Feb. 2012	26	26	0	0	2217.3	1081.6	51.2	828
Mar. 2012	27	27	0	0	2246.2	1003.4	55.3	960
Apr. 2012	28	28	0	0	2167.4	959.1	55.7	911
May 2012	29	29	0	0	2550.1	1189.8	53.3	1035
June 2012	30	30	0	0	2243.0	996.3	55.6	1019
July 2012	31	31	0	0	2263.6	1027.4	54.6	1140
Aug. 2012	32	32	0	0	2245.7	1015.0	54.8	1057
Sep. 2012	33	33	0	0	2459.3	1211.7	50.7	1064
Oct. 2012	34	34	0	0	2260.9	1037.9	54.1	1041
Nov. 2012	35	35	0	0	2380.8	1115.4	53.1	1077
Dec. 2012	36	36	0	0	2244.2	989.7	55.9	1041
Jan. 2013	37	37	0	0	3340.6	1702.5	49.0	963
Feb. 2013	38	38	0	0	3274.3	1656.1	49.4	564
Mar. 2013	39	39	0	0	3231.4	1677.5	48.1	888
Apr. 2013	40	40	0	0	3314.3	1605.7	51.6	931
May 2013	41	41	1	1	3863.9	2117.3	45.2	725
June 2013	42	42	1	2	3461.8	1954.2	43.5	729
July 2013	43	43	1	3	3907.3	2146.2	45.1	925
Aug. 2013	44	44	1	4	3448.6	1919.8	44.3	905
Sep. 2013	45	45	1	5	3689.0	2030.8	44.9	773
Oct. 2013	46	46	1	6	3691.9	2048.7	44.5	1010
Nov. 2013	47	47	1	7	3285.1	1790.1	45.5	1051
Dec. 2013	48	48	1	8	3486.2	1892.4	45.7	1135
Jan. 2014	49	49	1	9	4016.0	2172.4	45.9	1067
Feb. 2014	50	50	1	10	3760.6	2037.1	45.8	677
Mar. 2014	51	51	1	11	3749.1	2025.6	46.0	1095
Apr. 2014	52	52	1	12	3836.3	2096.7	45.3	984
May 2014	53	53	1	13	3904.6	2100.3	46.2	1004
June 2014	54	54	1	14	4028.7	2212.2	45.1	1056
July 2014	55	55	1	15	3911.0	2173.0	44.4	1170
Aug. 2014	56	56	1	16	4034.8	2244.4	44.4	1032
Sep. 2014	57	57	1	17	4136.9	2270.9	45.1	1071
Oct. 2014	58	58	1	18	4153.6	2300.6	44.6	1025
Nov. 2014	59	59	1	19	4136.7	2313.0	44.1	1016
Dec. 2014	60	60	1	20	4254.8	2330.0	45.2	1397
Jan. 2015	61	61	1	21	3673.4	1996.6	45.6	898
Feb. 2015	62	62	1	22	3857.2	2167.3	43.8	997
Mar. 2015	63	63	1	23	3834.0	2140.5	44.2	1388
Apr. 2015	64	64	1	24	4005.1	2168.1	45.9	1207
May 2015	65	65	1	25	3969.7	2154.0	45.7	1190
June 2015	66	66	1	26	3928.5	2114.2	46.2	1260
July 2015	67	67	1	27	3966.8	2198.5	44.6	1407
Aug. 2015	68	68	1	28	3918.8	2216.5	43.4	1328
Sep. 2015	69	69	1	29	4077.6	2328.7	42.9	1200
Oct. 2015	70	70	1	30	3892.7	2193.2	43.7	1160
Nov. 2015	71	71	1	31	4015.3	2265.3	43.6	1188
Dec. 2015	72	72	1	32	4153.8	2367.1	43.0	1368
Jan. 2016	73	73	1	33	3734.3	2201.7	41.0	1359
Feb. 2016	74	74	1	34	3519.7	1988.5	43.5	1201
Mar. 2016	75	75	1	35	3764.9	1996.9	47.0	1523
Apr. 2016	76	76	1	36	3830.5	1919.4	49.9	1152
May 2016	77	77	1	37	4106.5	2059.6	49.8	1165
June 2016	78	78	1	38	3911.4	2015.6	48.5	1178
July 2016	79	79	1	39	3824.6	1937.5	49.3	1292
Aug. 2016	80	80	1	40	3748.3	1926.7	48.6	1288
Sep. 2016	81	81	1	41	3837.0	1955.5	49.0	1095
Oct. 2016	82	82	1	42	3829.5	1926.1	49.7	1007
Nov. 2016	83	83	1	43	4005.1	2078.4	48.1	1145
Dec. 2016	84	84	1	44	3832.3	1926.9	49.7	1466

^1^ THE: total hospitalization expenses, ^2^ OOP: Out-of-pocket, ^3^ RR: reimbursement ratio.
